# Safety analysis of laboratory parameters in paediatric patients with spinal muscular atrophy treated with nusinersen

**DOI:** 10.1186/s12887-024-04955-0

**Published:** 2024-07-25

**Authors:** Xiaomei Zhu, Hui Li, Chaoping Hu, Min Wu, Shuizhen Zhou, Yi Wang, Wenhui Li

**Affiliations:** 1 Department of Neurology, Children’s Hospital of Fudan University, National Children’s Medical Center, Shanghai, China; 2 Department of Rehabilitation, Children’s Hospital of Fudan University, National Children’s Medical Center, Shanghai, China

**Keywords:** Nusinersen, Side effects, Laboratory parameters, Spinal muscular atrophy, Safety

## Abstract

**Background:**

Spinal muscular atrophy (SMA) is a progressive neurodegenerative disorder that can be treated with intrathecal nusinersen, an antisense oligonucleotide. In addition to efficacy, safety is a determining factor in the success of any therapy. Here, we aim to assess the safety of nusinersen therapy in paediatric patients with SMA.

**Methods:**

Laboratory data of paediatric patients with SMA who received nusinersen between October 2019 and May 2022 were retrospectively analysed.

**Results:**

During the observation period, 46 infants and children aged 2.9 months to 13.6 years received a total of 213 nusinersen doses without safety concerns. Inflammatory markers were stable throughout the study. International normalized ratio was increased by 0.09 per injection. Urea levels were increased by 0.108 mmol/L, and cystatin C decreased by 0.029 mg/L per injection. There were no significant changes in platelet count, activated partial thrombin time, creatinine levels or liver enzyme levels during treatment. The cerebrospinal fluid (CSF) leukocyte count remained stable, and total protein increased by 24.038 mg/L per injection.

**Conclusion:**

Our data showed that nusinersen therapy is generally safe in children with SMA. Laboratory monitoring did not identify any persistent or significantly abnormal findings. CSF protein should be monitored to gain more insights.

## What this paper adds

- Nusinersen therapy is generally safe in pediatric patients with SMA.
- No signs of clinically relevant platelet declines, coagulopathies, or kidney/liver toxicities.
- Inflammatory markers were stable throughout the study.
- No markedly abnormal findings that were persistent or led to altered treatment.
- CSF protein should be followed-up to gain more insights.

## Introduction

Spinal muscular atrophy (SMA) is an autosomal recessive neuromuscular disorder characterized by progressive muscular atrophy and weakness [1]. The most common form of SMA is caused by a homozygous deletion or mutation in the survival of the motor neuron 1 (*SMN1*) gene located on chromosome 5q. This leads to decreased levels of SMN protein expression and degeneration of motor neurons in the anterior horn cells of the spinal cord and brain stem [2].

SMA is classified based on the age of onset and motor milestones achieved during childhood, ranging from severely affected infants with onset before birth (type 0) and who never achieved sitting ability (type 1) to children who were unable to walk independently (type 2) and to a mild form manifesting in ambulatory individuals with onset in childhood (type 3) and with onset in adulthood (type 4) [3]. Nusinersen is an antisense oligonucleotide (ASO) that modifies the splicing of *SMN2* pre-mRNA, resulting in increased levels of full-length SMN protein [4]. Previous clinical trials have shown that treatment with intrathecal nusinersen yielded meaningful benefits in terms of motor functions in infants and children with infantile and later-onset SMA [5, 6]. A safety analysis [7] integrating seven clinical trials revealed that nusinersen was generally well tolerated without treatment-related serious adverse events; laboratory abnormalities, thrombocytopenia, and proteinuria were mostly mild in severity. All these findings were from clinical trials, and the data from real-world studies are deficient, especially the data regarding children.

Here, we present systematic data on laboratory findings in paediatric patients with SMA who were treated with nusinersen. These data should provide useful information for clinicians to assess the safety of nusinersen therapy in the paediatric population.

## Methods

Data from 46 paediatric patients with 5q SMA type 1, 2, or 3 who were treated with nusinersen (defined as at least one successful injection) in the Department of Neurology at the Children’s Hospital of Fudan University were retrospectively analysed.

Intrathecal injection of nusinersen was performed according to the recommended dosing schedule, with four loading doses on days 0 (V1 = baseline), 14 (V2), 28 (V3), and 63 (V4), followed by maintenance doses every four months (V5, V6…). Each dose was 12 mg (5 mL) nusinersen. Before injections, 5 mL of cerebrospinal fluid (CSF) was removed, and blood and urine samples were collected for laboratory analyses according to the standard of care. CSF analysis was added as standard care from January 2021 onwards.

The following laboratory parameters, collected at baseline and before each injection, were considered in the statistical evaluation: white blood cell (WBC) and platelet count, CRP, activated partial thrombin time (APTT), international normalized ratio (INR), fibrinogen, total bilirubin (TBIL), direct bilirubin (DBIL), alanine aminotransferase (ALT), aspartate transaminase (AST), alkaline phosphatase (ALP), gamma-glutamyl transferase (GGT), urea, creatinine, cystatin C, phosphocreatine kinase (CK); urinary WBC count, red blood cell (RBC) count and urinary protein; CSF parameters included WBC count, glucose and total protein.

### Reference values

The reference values for blood cell count and liver and kidney function were selected based on the patient age group as per the health industry standards of the People's Republic of China WS/T 779–2021 Reference Range for the Blood Cell Analysis of Children and the WS/T 780–2021 Reference Range for Common Clinical Biochemical Testing Items for Children [8, 9], respectively. The reference intervals of other parameters were as follows: CRP < 8 mg/L, APTT 26–40 s, INR 0.8–1.2, fibrinogen 2–4 g/L, TBIL 3.4–17.1 µmol/L, DBIL 0–6 µmol/L, cystatin C 0.55–1.55 mg/L, CK 0–164 U/L, CSF WBC 0–15*10^6^/L, CSF glucose 2.5–4.4 mmol/L, CSF total protein < 450 mg/L, urinary WBC 0–5 cells/high power field (HPF), urinary RBC 0–3/HP, and urinary protein negative.

### Statistical analysis

Statistical analyses were performed with IBM SPSS Statistics 25 (SPSS IBM, Armonk, NY, USA). Age was described by the mean, standard deviation, and range at baseline. To detect potential toxicities during nusinersen treatment, abnormal laboratory values that deviated above or below the normal range were calculated at baseline, and only medically reasonable or meaningful directions of deviation were considered. To identify trends in laboratory parameters, the change from baseline was calculated for continuous data by subtracting the baseline value from the value obtained preinjection at each treatment visit. Linear regression analysis was performed using the number of nusinersen doses as a dependent variable. Patients without available baseline data were excluded from the regression analysis. A two-sided *p* value < 0.05 was interpreted as significant.

## Results

### Patients and treatment characteristics

**Fig. 1:**
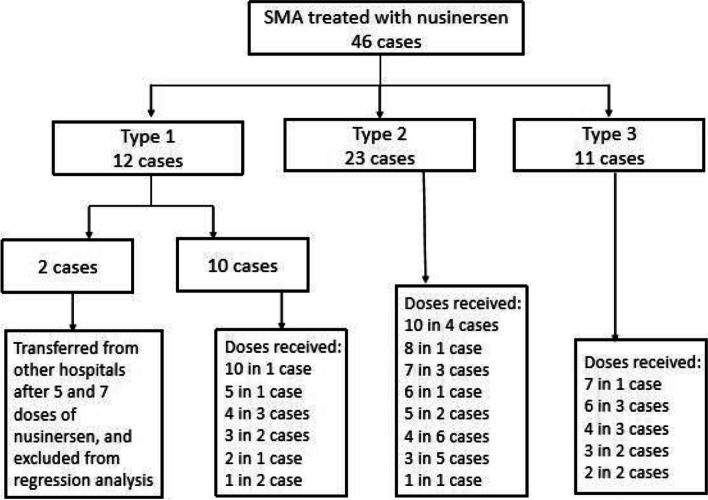
SMA types and doses received. SMA, spinal muscular atrophy

Between October 2019 and May 2022, a total of 46 paediatric patients with SMA were treated with nusinersen. There were a total of 213 successful injections (Fig. 1), with a median of four doses per patient (range 1–10) and a median treatment time of 2 months (range 0–26). Among these, 12 patients had SMA type 1, 23 patients had SMA type 2, and 11 patients had SMA type 3 (Table 1). Two patients who were transferred from other hospitals had received five and seven doses of nusinersen. They were excluded from the regression analysis but included in the calculation of deviation from the normal range.

**Table 1:** Subject characteristics at baseline

	**SMA Type 1**	**SMA Type 2**	**SMA Type 3**	**Total**
Number of patients	12	23	11	46
Female (%)	4 (33.3)	11 (47.8)	6 (54.5)	21 (45.7)
Mean age at onset, months (range)	3.1 (0.4–10.0)	8.8 (4.0–18.0)	34.1 (12.0–120.0)	13.4 (0.4–120.0)
Nusinersen starting age, years ± SD (range)	2.4 ± 1.9 (0.4–5.8)	4.7 ± 3.3 (0.7–10.4)	7.4 ± 3.1 (2.7–13.6)	4.7 ± 3.5 (0.4–13.6)

*SD* standard deviation

### Sample characteristics (Table 2)

**Table 2:** Deviation from the normal range on baseline

**Parameter** **, ** ***n/N*** ** (%)**		**SMA Type 1 ** **(** ***N*** ** = 12)**	**SMA Type 2 ** **(** ***N*** ** = 23)**	**SMA Type 3 ** **(** ***N*** ** = 11)**	**Total ** **(** ***N*** ** = 46)**
**Blood**					
WBC	**↑**	2/10	2/23	2/11	6/44
	**↓**	0/10	0/23	0/11	0/44
CRP	**↑**	0/10	0/23	1/10	1/44
	**↓**	NA	NA	NA	
Platelet	**↑**	1/10	2/23	1/11	4/44
	**↓**	0/10	0/23	0/11	0/44
INR	**↑**	0/10	0/23	0/11	0/44
	**↓**	NA	NA	NA	
APTT	**↑**	3/10	1/23	4/11	8/44
	**↓**	NA	NA	NA	
Fibrinogen	**↑**	0/10	0/23	1/11	1/44
	**↓**	2/10	3/23	0/11	5/44
Urea	**↑**	1/10	0/23	1/11	2/44
	**↓**	0/10	0/23	1/11	1/44
Creatinine	**↑**	0/10	0/23	0/11	0/44
	**↓**	6/10	16/23	9/11	31/44
Cystatin C	**↑**	0/8	0/18	0/9	0/35
	**↓**	1/8	4/18	2/9	7/35
CK	**↑**	0/12	9/23	5/11	14/46
	**↓**	NA	NA	NA	
TBIL	**↑**	0/10	0/23	1/11	1/44
	**↓**	NA	NA	NA	
DBIL	**↑**	0/10	0/23	0/11	0/44
	**↓**	NA	NA	NA	
ALP	**↑**	0/10	0/22	0/11	0/43
	**↓**	0/10	5/22	2/11	7/43
ALT	**↑**	2/10	1/23	2/11	5/44
	**↓**	0/10	0/23	0/11	0/44
AST	**↑**	3/10	1/23	2/11	6/44
	**↓**	0/10	0/23	0/11	0/44
GGT	**↑**	1/10	0/22	0/10	1/42
	**↓**	0/10	0/22	0/10	0/42
**Urine**					
Protein	**↑**	0/9	1/23	1/11	2/43
	**↓**	NA	NA	NA	
WBC	**↑**	0/9	1/23	0/11	1/43
	**↓**	NA	NA	NA	
RBC	**↑**	0/9	0/23	0/11	0/43
	**↓**	NA	NA	NA	
**CSF**					
WBC	**↑**	0/6	0/15	0/9	0/30
	**↓**	NA	NA	NA	
Glucose	**↑**	NA	NA	NA	
	**↓**	0/6	0/15	0/9	0/30
Protein	**↑**	0/6	0/15	0/9	0/30
	**↓**	NA	NA	NA	

*ALP* alkaline phosphatase, *ALT* alanine aminotransferase, *APTT* activated partial thromboplastin time, *AST* aspartate aminotransferase, *CK* phosphocreatine kinase, *CRP* C-reactive protein, *CSF* cerebrospinal fluid, *DBIL* direct bilirubin, *GGT* gamma-glutamyltransferase, *INR* international normalized ratio, *n* number of abnormal values above or below the limit of normal range, *N* denominator of the data which represents the available sample number of each parameter at baseline, *NA* not applicable (only medically reasonable or meaningful directions of deviation were considered), *RBC* red blood cell, *TBIL* total bilirubin, *WBC* white blood cell

Blood counts, kidney and liver values, and coagulation parameters were available for all 213 injections. The total number of measurements collected was 212 for ALP (99.5%), 212 for CRP (99.5%), 207 for GGT (97.2%), 171 for cystatin C (80.3%), and 164 for CK (77%). Eight of 156 available CSF samples (including three samples at baseline) from seven patients were excluded from statistical evaluation due to blood contamination, leaving a total of 148 samples (69.5%) available for further evaluation. The total number of urinary parameters available was 211 for cell counts and protein (99.1%).

### Laboratory analysis—baseline values and changes during treatment with nusinersen

The number of abnormal values in baseline parameters is provided in Table 2. The number of abnormal laboratory values that might indicate drug toxicity in individual patients during nusinersen treatment is shown in Table 3. The results from linear regression analysis of laboratory value changes are shown in Table 4.

**Table 3:** Shifts to abnormal laboratory values*

Parameters, *n/N*(%)	SMA type 1 *N* = 12	SMA type 2 *N* = 23	SMA type 3 *N* = 11	Total *N* = 46
High blood WBC	0 (0.0%)	3 (13.0%)	0 (0.0%)	3 (6.5%)
High CRP	0 (0.0%)	3 (13.0%)	0 (0.0%)	3 (6.5%)
Low platelet	0 (0.0%)	0 (0.0%)	1 (9.1%)	1 (2.2%)
High INR	0 (0.0%)	0 (0.0%)	0 (0.0%)	0 (0.0%)
High APTT	0 (0.0%)	7 (30.4%)	3 (27.3%)	10 (21.7%)
High blood urea	0 (0.0%)	0 (0.0%)	0 (0.0%)	0 (0.0%)
High blood creatinine	0 (0.0%)	0 (0.0%)	1 (9.1%)	1 (2.2%)
High cystatin C	0 (0.0%)	0 (0.0%)	0 (0.0%)	0 (0.0%)
Urinary protein positive	1 (8.3%)	1 (4.4%)	0 (0.0%)	2 (4.3%)
High TBIL	0 (0.0%)	0 (0.0%)	0 (0.0%)	0 (0.0%)
High DBIL	0 (0.0%)	0 (0.0%)	0 (0.0%)	0 (0.0%)
High ALP	0 (0.0%)	1 (4.4%)	0 (0.0%)	1 (2.2%)
High ALT	1 (8.3%)	0 (0.0%)	0 (0.0%)	1 (2.2%)
High AST	1 (8.3%)	2 (8.7%)	0 (0.0%)	3 (6.5%)
High GGT	1 (8.3%)	0 (0.0%)	0 (0.0%)	1 (2.2%)
High CSF WBC	0 (0.0%)	0 (0.0%)	0 (0.0%)	0 (0.0%)
Low CSF glucose	0 (0.0%)	0 (0.0%)	0 (0.0%)	0 (0.0%)
High CSF protein	0 (0.0%)	1 (4.4%)	0 (0.0%)	1 (2.2%)

*ALP* alkaline phosphatase, *ALT* alanine aminotransferase, *APTT* activated partial thromboplastin time, *AST* aspartate aminotransferase, *CRP* C-reactive protein, *CSF* cerebrospinal fluid, *CSF* cerebrospinal fluid, *DBIL* direct bilirubin, *GGT* gamma-glutamyltransferase, *INR* international normalized ratio, *n* number of abnormal values above or below the limit of normal range, *N* denominator of the data that represents the number of each SMA type group, *SMA* spinal muscular atrophy, *TBIL* total bilirubin, *WBC* white blood cell

^*^ This tables showed the shifts to abnormal values in those patients with normal baseline values

**Table 4:** Details of linear regression analysis base on nusinersen doses

Dependent value	Coefficient	*t*	*p* value	R^2^	Adjusted R^2^	F
Blood						
WBC	0.120	1.632	0.105	0.017	0.010	2.663
Platelet	-0.392	-0.151	0.880	0.000	-0.006	0.023
**Urea**	**0.108**	**2.404**	**0.017**	**0.035**	**0.029**	**5.780**
Creatinine	-0.217	-0.918	0.360	0.005	-0.001	0.842
**Cystatin C**	**-0.026**	**-4.993**	**0.000**	**0.193**	**0.186**	**24.932**
TBIL	0.212	0.926	0.356	0.005	-0.001	0.858
DBIL	0.030	1.359	0.176	0.012	0.005	1.847
ALT	-0.019	-0.025	0.980	0.000	-0.006	0.001
AST	-0.570	-0.926	0.356	0.005	-0.001	0.858
GGT	0.053	0.359	0.720	0.001	-0.006	0.129
ALP	-0.178	-0.071	0.943	0.000	-0.007	0.005
CK	4.631	1.767	0.080	0.029	0.020	3.123
**INR**	**0.009**	**2.796**	**0.006**	**0.047**	**0.041**	**7.820**
APTT	0.073	0.576	0.565	0.002	-0.004	0.332
Fibrinogen	0.036	1.801	0.074	0.020	0.014	3.244
CSF						
WBC	1.500	5.196	0.121	0.964	0.929	27.000
**Glucose**	**0.436**	**8.868**	**0.000**	**0.464**	**0.458**	**78.637**
**Protein**	**24.038**	**6.013**	**0.000**	**0.256**	**0.249**	**36.153**
Urine						
RBC	0.037	0.490	0.625	0.002	-0.005	0.240
WBC	-0.233	-0.709	0.480	0.003	-0.003	0.502

*ALP* alkaline phosphatase, *ALT* alanine aminotransferase, *APTT* activated partial thromboplastin time, *AST* aspartate aminotransferase, *CK* phosphocreatine kinase, *CSF* cerebrospinal fluid, *DBIL* direct bilirubin, *GGT* gamma-glutamyltransferase, *INR* international normalized ratio, *RBC* red blood cell, *TBIL* total bilirubin, *WBC* white blood cell

#### Inflammatory markers

WBC count was elevated in six of 44 available baseline samples, with a maximum value of 15.77*10^9^/L. In one of 44 available baseline samples, CRP was slightly elevated; the value was 11 mg/dL. Regression analysis showed no significant change in WBC count (*p* = 0.105) during treatment with nusinersen.

Among SMA type 1 patients, one patient whose WBC count was slightly elevated at baseline eventually had a normal count before dose V4, and the other patient whose WBC count was slightly elevated did not come back for further treatment. Among SMA type 2 patients, two patients had slightly elevated WBC count at baseline, but both eventually had a normal count before dose V3, two patients had a slightly high WBC count on the last visit, and another patient had transient and slight elevation of WBC count at several time points. No patient with SMA type 3 had a high WBC count at any visit. All patients with SMA types 1 and 3 had normal CRP levels. In three patients with SMA type 2, CRP levels showed a transient increase to 19.73 mg/dL, possibly indicating mild systemic infection.

#### Platelets

At baseline, there were four patients with elevated platelets at a maximum of 633*10^9^/L, and no patients had low platelet counts. While on treatment with nusinersen, only one patient had a low platelet count, which was 101*10^9^/L at dose V3; the count increased to the normal range at dose V4. There was no significant change (*p* = 0.880) in platelet count during the observation period according to regression analysis.

#### Coagulation parameters

At baseline, there were no patients with a high INR, and eight patients had slightly elevated APTT, ranging from 40.7–44.7 s (median 43.0). During nusinersen treatment, there were no patients with a high INR, while 14 patients had transient and slight elevation of APTT, ranging from 40.4–47.6 s (median 42.1). Of these 14 patients, four had high APTT at baseline and at the loading doses, but these eventually normalized without intervention; three had transiently elevated APTT, but these eventually normalized without intervention; six showed high APTT at the last visit; and another had high APTT at the last two visits as of data cutoff.

Regression analysis showed a significant change (*p* = 0.006) in INR during the observation period, with INR increasing by 0.09 per injection. The APTT change was not significant during the observation period (*p* = 0.565).

#### Kidney values and urinary analysis

At baseline, there were 31 patients with low creatinine levels, two with extremely low urinary protein levels and one with a slightly elevated urinary WBC count; no patients had haematuria. Regarding urea levels, two patients showed slightly increased values, while one had a low value. Seven patients had low cystatin C values (range 0.42–0.54 mg/L), and no patients had high readings.

During treatment, one patient with SMA type 3 showed high creatinine levels at V3 and V4, 52.6 and 71.7 µmol/L respectively (reference range is 19–44 umol/L), which were decreased to the normal range without intervention at the V5 follow-up (21.6 umol/L) under sequential nusinersen treatment. The blood pressure, urinalysis, cystatin C level and urinary system ultrasound were all normal. Only one patient had slightly high urea levels during treatment but this patient also had a high urea level at baseline, and no patients had high cystatin C levels. Two patients with extremely low urinary protein levels at baseline eventually exhibited a negative shift in those levels during treatment with nusinersen. Another two patients showed extremely low levels of urinary protein while under treatment, but these episodes were transient. Transient haematuria was observed in ten patients, nine of whom had RBC counts as high as 4/HP and one of whom had an RBC count as high as 21/HP. Seven patients had transient increases in urinary WBC counts to a range of 10–102/HP, which eventually normalized without antibiotic treatment. Abnormal urinary WBC counts in two patients were due to contamination during sample collection, and they were excluded from regression analysis.

Regression analysis of urea levels showed a significant change (*p* = 0.017) during the observation period. Urea levels increased by 0.108 mmol/L per injection. There were no significant changes in urinary WBC, RBC, and creatinine levels (*p* > 0.05). For cystatin C levels, regression analysis showed a significant change (*p* = 0.000) during nusinersen treatment, with a decrease of 0.029 mg/L per injection.

#### Liver values

At baseline, all patients had a normal DBIL level, and one had a slightly elevated TBIL value. No patients had elevated ALP, while seven patients had decreased ALP values to a range of 82.7–142.0 U/L. Regarding GGT, only one patient had a slightly elevated value (40.32 U/L), and no patients had a value below the reference range. Regarding ALT, five patients had elevated values, which were as high as 133.94 IU/L, but 4 of them eventually had normal or nearly normal values from the V2 or V5 follow-up onwards; the one remaining patient had not been re-examined as of the data cutoff date. Regarding AST, elevated values (as high as 124.01 IU/L) were obtained in six patients, three of whom eventually decreased to normal or nearly normal values at the V2 to V5 follow-up. One patient continued to have slightly elevated values at V2, and two were not re-examined as of the data cutoff date.

During treatment with nusinersen, no patients had elevations in TBIL or DBIL. Only one patient with SMA type 2 had a transient elevation in ALP value up to 939 IU/L. Another patient with SMA type 1 had a slightly elevated GGT value at 21.18 IU/L. Among the patients with normal ALT or AST at baseline, one had an elevated ALT, which was 66.98 IU/L at V2, and was not re-examined as of the data cutoff date, one had transient and slightly elevated AST, and another two patients showed elevated AST (as high as 83.27 IU/L) at the last visit as of the data cutoff date.

Regression analysis showed no significant changes in TBIL, DBIL, ALP, GGT, ALT, and AST values (*p* > 0.05) during the observation period.

#### CSF parameters

At baseline, all patients with available CSF data had normal WBC counts and normal glucose levels. Total protein levels were also normal, except for one patient with SMA type 3 who had elevated CSF total protein (681 mg/L) that decreased slightly (to 675 mg/L) at the V2 cutoff date. One patient with SMA type 2 had a transient high CSF protein level (498.8 mg/L) at V9. Both of them had no clinical symptoms of CNS infection and hydrocephalus. While patients were under treatment with nusinersen, WBC count and glucose and protein levels in CSF in all other patients remained within the normal range.

Regression analysis revealed no significant changes in CSF WBC count (*p* > 0.05) during the observation period. In contrast, CSF glucose levels changed significantly during the observation period (*p* = 0.000), increasing by 0.436 mmol/L per injection. CSF total protein levels also changed significantly during the observation period (*p* = 0.000), with an increase of 24.038 mg/L per injection.

### Clinical adverse events

Adverse events after lumbar puncture (LP) procedure and intrathecal injection were available for 213 LPs. Post lumbar puncture headache was observed in 3 of 213 LPs (1.4%). Back pain was reported for 4 LPs(1.9%). Nausea was reported for 9 LPs (4.2%) and vomiting for 8 LPs (3.8%). Those events developed in 1–3 days after lumbar puncture procedure and recovered in one week without hospitalization. No symptoms of hydrocephalus were reported, such as rapidly increasing head circumference, continuous headache, visual impairment or balance disorder.

## Discussion

This safety analysis of data from 46 infants and children with symptomatic SMA showed no evidence of systemic toxicities with nusinersen treatment, such as thrombocytopenia, increased aminotransferase, and proteinuria, which have previously been observed with certain ASOs [10]. This may be partly attributed to the intrathecal administration, dose, and dosing frequency of nusinersen; related differences from other ASOs lead to a lower level of systemic exposure [7].

In a real-world study, there was no further evidence of an increased risk of severe thrombocytopenia in children treated with nusinersen. There have been reports of moderate to severe thrombocytopenia with other ASOs, such as drisapersen (BioMarin Pharmaceuticals) and the telomerase inhibitor imetelstat (Geron) [11, 12]. In the ENDEAR study, the incidence of shifts to low platelet counts was higher among nusinersen-treated infants (13% vs. 0%), and the incidence was lower among nusinersen-treated children in the CHERISH study (20% vs. 26%) compared with controls [7]. Platelet counts < 20 × 10^9^/L occurred in one infant in the ENDEAR study and two children in the CHERISH study, while these patients eventually had normal or only isolated low platelet counts at later assessments [7]. There was only a single case of sporadic thrombocytopenia with a platelet count of 101*10^9^/L in our analysis, and platelet levels remained stable over time, consistent with adult data from another study [13].

Although all INR values were within the normal range, an elevation in INR during nusinersen treatment according to the regression analysis should be under observation afterwards. Some studies on the relationship between antisense oligonucleotide and INR, including Mipomersen (a subcutaneous injectable apolipoprotein B-100 synthesis inhibitor) and IONIS-FXIRx (2nd generation antisense oligonucleotide that specifically reduces human FXI mRNA expression in the liver), showed no clinically meaningful differences in INR [14, 15]. Similar to the report by Goedeker et al. [16], there were 14 patients in our cohort who had slight and transient elevation in APTT values. However, this was not significant by regression analysis. Longer follow-up is needed to observe the trends in INR and APTT during nusinersen treatment.

In our study, there were no reports of renal failure, glomerulonephritis, or nephrotic syndrome in participants receiving nusinersen. In our cohort, baseline creatinine levels were low in most patients (70%), and these levels did not change significantly during nusinersen treatment. This is consistent with previous studies [17–19], which also found low creatinine levels in SMA patients. Creatinine levels were temporarily high at V3 and V4, and returned to normal at V5 and V6 in one boy. At nine months old, he was found to have hydronephrosis in the left kidney. While during the nusinersen therapy, the urinary tract ultrasound showed normal. The temporarily high creatinine levels in the boy could not be explained very clearly, and we should pay more attention on it. Because low creatinine levels are typical in SMA, the glomerular filtration rate might be overestimated, and cystatin C levels would be a good supplementary indicator. In terms of cystatin C levels, our analysis showed normal or low values at baseline that decreased slightly by 0.029 mg/L per injection during nusinersen according to regression analysis. In the CHERISH and ENDEAR studies, few patients had high cystatin C levels in the nusinersen-treated group (0–1%) and the control-treated group (0–3%) [7]. There were only 2 patients with transient extremely low urinary protein during nusinersen treatment in our study, which was consistent with previous clinical trials [6, 7].

To date, there has been no evidence of clinically relevant hepatotoxicity from treatment with nusinersen. In our study, there were 5 and 6 of 44 patients with elevated ALTs and ASTs, respectively, and normal bilirubin at baseline. Elevated ALTs in 4 cases and ASTs in 5 cases decreased without any symptomatic treatments. No clear patterns were observed in the incidence of shifts to high ALT or high AST in the clinical trials of nusinersen [7]. The NURTURE trial showed that transaminase levels were stable in presymptomatic children treated with nusinersen [20]. The transient elevation in ALT and AST levels in our study might be partly attributed to the pathology of SMA.

Although only one patient experienced elevated CSF total protein at V9 in our analysis, regression analysis showed a significant increase in CSF total protein in paediatric patients with nusinersen. Previously, there were no safety concerns regarding CSF parameters such as total cell count and levels of protein, glucose, inflammatory cytokines, and antinusinersen antibodies in phase 1 and 2 clinical trials [5, 21]. On the other hand, elevation of total CSF protein levels has been reported in several studies focused on adolescent and adult SMA patients receiving nusinersen treatment [22–24]. A potential impact of repeated lumbar puncture on CSF total protein elevation was suggested by two studies [22, 23]**.** Although there were no clinical signs of hydrocephalus in our study, we should pay attention to long-term CSF protein changes and the clinical manifestation related to hydrocephalus.

Our study has some limitations. Given the limited cohort size and short observation period, some side effects may not have been captured. In addition, it is possible that the transient abnormal findings that occurred could reappear. CSF sample collection was not initiated with the first patient.

## Conclusion

Our data demonstrate that nusinersen therapy is generally safe in paediatric patients with SMA. There was no evidence of clinically relevant platelet decline, coagulopathy, or renal or hepatic organ toxicity, which are common concerns with the use of ASOs. Frequent serial laboratory monitoring might not identify any significantly abnormal findings, and CSF protein should be monitored to gain more insights.

## Data Availability

The datasets used and/or analysed during the current study are available from the corresponding author upon reasonable request.

## Abbreviations

ALP: Alkaline phosphatase
ALT: Alanine aminotransferase
APTT: Activated partial thrombin time
ASO: Antisense oligonucleotide
AST: Aspartate transaminase
CK: Phosphocreatine kinase
CRP: C-reactive protein
CSF: Cerebrospinal fluid
DBIL: Direct bilirubin
GGT: Gamma-glutamyl transferase
HP: High power field
INR: International normalized ratio
RBC: Red blood cell
SD: Standard deviation
SMA: Spinal muscular atrophy
*SMN 1*: Survival of motor neuron 1
TBIL: Total bilirubin
WBC: White blood cell
